# Genome-Wide Identification and Characterization of the JAZ Gene Family in Rubber Tree (*Hevea brasiliensis*)

**DOI:** 10.3389/fgene.2019.00372

**Published:** 2019-05-01

**Authors:** Jinquan Chao, Yue Zhao, Jie Jin, Shaohua Wu, Xiaomin Deng, Yueyi Chen, Wei-Min Tian

**Affiliations:** ^1^Ministry of Agriculture Key Laboratory of Biology and Genetic Resources of Rubber Tree/State Key Laboratory Breeding Base of Cultivation and Physiology for Tropical Crops, Rubber Research Institute, Chinese Academy of Tropical Agricultural Sciences, Haikou, China; ^2^College of Agronomy and Biotechnology, China Agricultural University, Beijing, China; ^3^Nextomics Biosciences Co., Ltd., Wuhan, China

**Keywords:** *Hevea brasiliensis* Muell. Arg., natural rubber biosynthesis, JAZ identification, laticifer differentiation, vascular cambium region

## Abstract

Jasmonate signaling plays a vital role in the regulation of secondary laticifer differentiation and natural rubber biosynthesis in *Hevea brasiliensis*. Jasmonate ZIM-domain (JAZ) proteins are the master regulators of jasmonate signaling. Although several JAZs have been reported in the laticifer cells of *H. brasiliensis*, the genome-wide screening of *HbJAZ* members has not yet been explored. In the present study, 18 *HbJAZs* were identified based on the recent *H. brasiliensis* genome. Phylogenetic construction revealed that the HbJAZs were clustered into five subgroups and that members within the same subgroup shared highly conserved gene structures and protein motifs. *Cis*-element analysis of *HbJAZ* promoters suggested the presence of hormone, stress and development-related *cis*-elements. HbJAZ1.0, HbJAZ2.0, and HbJAZ5.0 interacted with CORONATINE INSENSITIVE1 (COI1) in the presence of coronatine (COR, a JA mimic). HbJAZ1.0, HbJAZ2.0, HbJAZ5.0, and HbJAZ12.0 could also interact with each other. Of the 18 *HbJAZs*, transcripts of 15 *HbJAZs* were present in the vascular cambium region except for that of *HbJAZ7.0*, *HbJAZ8.0d*, and *HbJAZ13.0*. Fourteen of the 15 *HbJAZs* were significantly up-regulated upon COR treatment. The transcripts of three genes that were absent from vascular cambium region were also absent from the latex. Among the 15 *HbJAZs* in the latex, the expression patterns of 13 *HbJAZs* were different between the tapping and ethrel treatments. Eight of the 14 COR-up-regulated *HbJAZs* in the vascular cambium region were also activated by tapping in latex. Of the eight tapping-activated *HbJAZs*, 5 *HbJAZs* were repressed by ethrel application. Based on the computational analyses and gene expression patterns described in this study, the HbJAZ5.0 and HbJAZ10.0b may be associated with laticifer differentiation while the HbJAZ8.0b is a negative regulator for natural rubber biosynthesis in *H. brasiliensis*.

## Introduction

Jasmonates regulate various biological processes by the COI1-JAZ-MYC signaling pathway (Wasternack and Hause, 2013; Song et al., 2014; Yuan and Zhang, 2015). As repressors of jasmonate signaling, Jasmonate ZIM-domain (JAZ) proteins interact with MYC in the absence of active jasmonate (Singh et al., 2015). They are degraded by COI1-mediated ubiquitination in the presence of active jasmonate. This degradation releases MYC, which activates downstream gene transcription (Fonseca et al., 2009; Wager and Browse, 2012). The released MYC in turn activates the transcription of JAZ (Zhang et al., 2015). The JAZ proteins are a subgroup of the TIFY family, characterized by the N-terminal located ZIM domain and the C-terminal located Jas domain (Thines et al., 2007; Melotto et al., 2008; Li et al., 2017). The ZIM domain, which consists of 30 amino acids with a highly conserved TIFY motif (TIF[F/Y]XG), is required for the formation of homo- and hetero-dimers within the JAZ proteins (Vanholme et al., 2007). The Jas domain, containing 29 amino acids with a conserved motif (SLX_2_FX_2_KRX_2_RX_5_PY), is specific to JAZ members and interacts with a wide range of other proteins, such as COI1, and members of the MYC, MYB, and bHLH families (Yan et al., 2007; Sheard et al., 2010; Pauwels and Goossens, 2011). As a master regulator of jasmonate signaling, JAZ has been shown to directly regulate plant morphology, flower initiation, cotton fiber initiation, salvianolic acids, and tanshinone biosynthesis (Boter et al., 2015; Hu et al., 2016; Pei et al., 2018; Yu et al., 2018).

As an important industrial raw material, natural rubber is mainly produced by the commercialized tropical plant, the rubber tree (*Hevea brasiliensis*) (Chao et al., 2016). The secondary laticifers located in the bark of the trunk of *H. brasiliensis* are the site closely related to natural rubber production (Hao and Wu, 2000). Latex, a milky cytoplasm of laticifers, contains 20–40% of the rubber and is used as the raw material for refining natural rubber (Chao et al., 2017; Zhang et al., 2017). Although application of ethrel (an ethylene releaser) causes a significant increase in rubber yield per tapping via prolonging the duration of latex flow (Zhu and Zhang, 2009), it inhibits rubber biosynthesis (Deng et al., 2018). Activation of jasmonate signaling in laticifer cells is closely associated with the tapping-enhanced natural rubber biosynthesis (Zhao, 2011; Cao et al., 2017; Deng et al., 2018). Moreover, jasmonates are the key signal molecules for the secondary laticifer differentiation from vascular cambia in *H. brasiliensis* (Hao and Wu, 2000; Tian et al., 2015; Wu et al., 2016).

Until now, the core JA signaling components, such as *HbCOI1* and *HblMYC1-2*, have been identified in the latex of *H. brasiliensis*, and a total of 10 *HbJAZs* have been reported in different studies (Peng et al., 2009; Tian et al., 2010; Zhao et al., 2011; Pirrello et al., 2014; Hong et al., 2015). The publication of several versions of the genome of *H. brasiliensis* (Rahman et al., 2013; Lau et al., 2016; Tang et al., 2016; Pootakham et al., 2017; Makita et al., 2018) facilitates the genome-wide identification of JAZ family members. In the present study, a total of 18 *HbJAZs* were identified on the basis of the latest version of the *H. brasiliensis* genome (Tang et al., 2016), analysis of the gene structures, phylogenetics, interactions, and expression patterns of HbJAZ family members were performed, and candidate members that may be related to laticifer differentiation and rubber biosynthesis were suggested.

## Materials and Methods

### Plant Materials and Treatments

Plantlets, 10-year-old virgin trees and regularly tapped trees of the *H. brasiliensis* clone CATAS7-33-97 were grown in the field of the Experimental Farm of the Chinese Academy of Tropical Agricultural Sciences (CATAS) on Hainan Island. The plantlets were developed from the latent buds on pruned trunks and thus called epicormic shoots. The COR treatment and collection of vascular cambia-containing samples of epicormic shoots were previously described in Wu et al. (2016). At each time interval, the bark tissues were collected from 10 epicormic shoots and combined as one sample. The regularly tapped trees were tapped by using a half spiral pattern every 3 days (S/2, d/3). The virgin trees had not been tapped until sampling. Application of 1.5% ethrel on the tapping panel of the regularly tapped rubber trees were performed. The latex samples were collected 24 h after ethrel treatment. The latex samples were also collected from regularly tapped rubber trees without ethrel treatment and virgin rubber trees. The latex samples from 10 tapped trees with or without ethrel treatment and 10 virgin trees were combined as one sample, respectively. Three biological replications were conducted.

### RNA Isolation, DNA Isolation, and cDNA Synthesis

Total RNA was extracted from samples using RNAplant Plus reagent and evaluated by the NanoDrop 2000 (Thermo Scientific, Inc., United States). Approximately, 1 μg of RNA was used for reverse transcription and cDNA was synthesized using RevertAid^TM^ First Strand cDNA Synthesis Kit (Thermo Scientific, Inc., United States). Genomic DNA was extracted from the leaves of epicormic shoots using DNAplant Plus reagent.

### Computational Analysis

The whole genome sequence of *H. brasiliensis* was downloaded from NCBI^1^ (Tang et al., 2016). The *Arabidopsis* JAZ (AtJAZ) proteins were acquired from the TAIR database^2^. The HMM profiles of the TIFY domain (PF06200) and Jas domain (PF09425) were used as queries to search for predicted JAZ proteins in the *H. brasiliensis* genome using HMMER software (HMMER^3^). The BLAST algorithm was also used to identify the predicted *H. brasiliensis* JAZ proteins using AtJAZ as the queries. All putative HbJAZ proteins were further confirmed by the CDD database^4^. The AtJAZs and HbJAZs were aligned using the online software Multiple Sequence Alignment^5^, and then the phylogenetic tree was constructed using iTOL online software^6^. The molecular weight and isoelectric points of the HbJAZ proteins were predicted from the ExPASy database^7^. The genes structures and protein motif analysis were performed by GSDS2.0 software^8^ and MEME software^9^, respectively. For MEME analysis, the number of motifs was set as 12. The identified motifs were annotated by the InterProScan database^10^. *Cis*-acting regulatory elements within the *HbJAZ* promoters were analyzed by Plantcare^11^. The 1,500 bp genomic sequences upstream of the ATG of the HbJAZ genes were downloaded from the published *H. brasiliensis* genomes and used for the *cis*-regulatory element analysis.

### Yeast Two-Hybrid Assay

The full-length CDSs of five HbJAZ proteins (HbJAZ1.0/2.0/5.0/10.0b/12.0) and HbCOI1 were amplified and inserted into the pGBKT7 vector to generate a bait plasmid. The CDS of HbJAZ genes were then fused into the pGADT7 vector to generate a prey plasmid. The bait vector and prey vector were transformed into Y187 and Y2H gold strains, respectively. The yeast Y187 and Y2H gold strains were mated, and protein interaction was assessed by the expression of different reporters under control of GAL4-responsive promoters. For the interactions between HbJAZs and HbCOI1, 20 or 60 μM COR and 10% ethanol were respectively added as treatment or MOCK, according to the reference (Melotto et al., 2008).

### qRT-PCR Analysis

The primers for the *HbJAZs* were designed using the Primer Premier 5 software (Supplementary Table S1). The experiment was performed with the CFX384 real-time PCR system (Bio-Rad, United States) using the SYBR Prime Script RT-PCR Kit (TaKaRa, Dalian). *HbUBC2b* was used as the standard control for gene normalization (Chao et al., 2016). Three biological replicates were performed.

### Statistical Analysis

The relative expression of *HbJAZs* were assessed by the 2^−ΔΔCt^ method. Significant differences in the expression level were tested by Student’s *t*-test using GraphPad Prism 5 software.

## Results

### Identification and Isolation of JAZ Family Proteins in *H. brasiliensis*

**FIGURE 1:**
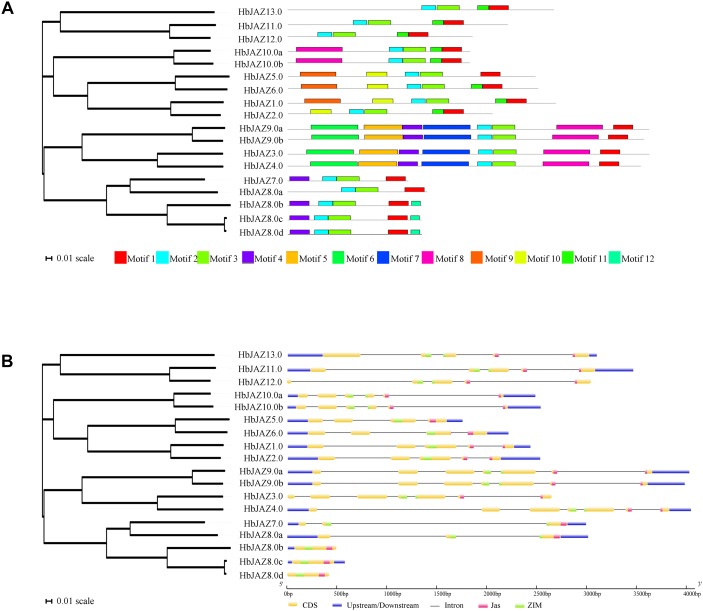
Structural analysis of HbJAZ family. **(A)** Motif analysis of of HbJAZ genes. 12 conserved motifs were identified using MEME program. **(B)** The exon/intron organization of HbJAZ genes. The yellow and blue boxes indicated coding sequence and untranslated region, respectively. Thin black lines indicated introns. The pink and green boxes respectively indicated Jas domain and ZIM domain.

The HMM profiles of the TIFY domain (PF06200) and Jas domain (PF09425), as well as BLAST searches using *Arabidopsis* JAZ sequences as queries, were used to perform genome-wide identification of JAZ proteins in *H. brasiliensis* based on the most recent genome database. After validating the TIFY domain and Jas domain by NCBI Conserved Domain Search Service, a total of 18 HbJAZ proteins were identified. The identified HbJAZs were designated based on the names of their presumptive *Arabidopsis* orthologs (Figure 1). The 18 HbJAZs ranged from 127 (HbJAZ7.0) to 382 (HbJAZ9.0a) amino acid residues in length, with molecular weights of 14.39 kDa (HbJAZ7.0) to 40.82 kDa (HbJAZ9.0a) (Table 1 and Supplementary Table S2). The predicted isoelectric points of these proteins ranged from 8.44 (HbJAZ7.0) to 9.91 (HbJAZ10.0b) (Table 1).

**Table 1:** Detail information on putative *HbJAZ* genes.

Gene name^a^	Gene symbol	Protein length (aa)	Protein Mw (kDa)	Protein pI	NCBI accession	Previously reported	At_ortholog^b^
HbJAZ1.0^∗^	scaffold0829_182974	283	30.59	9.52	XM_021788791.1	NA	AtJAZ1 (95%)
HbJAZ2.0	scaffold0609_18761	216	23.35	9.82	XM_021780844.1	HbJAZ1 (Tian et al., 2010)	AtJAZ2 (82%)
HbJAZ3.0	scaffold3142_11732	382	40.80	9.4	XM_021807489.1	HbJAZ_17062 (Pirrello et al., 2014)	AtJAZ3 (87%)
HbJAZ4.0^∗^	scaffold1173_227117	374	39.76	9.48	XM_021796925.1	NA	AtJAZ4 (91%)
HbJAZ5.0^∗^	scaffold0665_304891	261	28.16	9.52	XM_021783192.1	NA	AtJAZ5 (88%)
HbJAZ6.0	scaffold0015_736848	265	28.86	9.08	XM_021824535.1	HbJAZ_19967 (Pirrello et al., 2014) HbJAZ2 (Hong et al., 2015)	AtJAZ6 (95%)
HbJAZ7.0^∗^	scaffold1016_1652	127	14.39	8.44	XM_021793636.1	NA	AtJAZ7 (98%)
HbJAZ8.0a	scaffold0724_3556	147	16.37	9.25	XM_021785013.1	HbJAZ_1405 (Pirrello et al., 2014) HbJAZ8 (Hong et al., 2015)	AtJAZ8 (72%)
HbJAZ8.0b^∗^	scaffold0506_554681	141	16.03	8.99	XM_021834813.1	NA	AtJAZ8 (92%)
HbJAZ8.0c	scaffold1016_17771	141	16.20	9.75	XM_021793630.1	HbJAZ7 (Hong et al., 2015)	AtJAZ8 (92%)
HbJAZ8.0d^∗^	scaffold1016_13049	141	16.15	9.62	XM_021793637.1	NA	AtJAZ8 (92%)
HbJAZ9.0a	scaffold0440_1012668	382	40.82	8.46	XM_021832067.1	HbJAZ9 (Hong et al., 2015)	AtJAZ9 (72%)
HbJAZ9.0b	scaffold0429_394272	376	40.22	8.80	XM_021831491.1	HbJAZ_2001 (Pirrello et al., 2014)	AtJAZ9 (72%)
HbJAZ10.0a	scaffold0887_176192	192	21.60	9.30	XM_021790353.1	HbJAZ_29511 (Pirrello et al., 2014) HbJAZ10 (Hong et al., 2015)	AtJAZ10 (91%)
HbJAZ10.0b^∗^	scaffold0166_117253	192	21.53	9.91	XM_021817371.1	NA	AtJAZ10 (91%)
HbJAZ11.0	scaffold0762_419876	232	24.56	8.63	XM_021786280.1	HbJAZ_1229 (Pirrello et al., 2014)	AtJAZ11 (50%)
HbJAZ12.0	scaffold0026_2677018	195	20.75	9.69	XM_021835876.1	HbJAZ_863 (Pirrello et al., 2014) HbJAZ11 (Hong et al., 2015)	AtJAZ12 (70%)
HbJAZ13.0^∗^	scaffold0042_2919125	281	31.41	9.67	XM_021794816.1	NA	NA

*NA, not applicable; ^a^asterisk represents the genes identified in this work; ^b^shows the best Arabidopsis ortholog, and percentage in the brackets indicate similarity index.*

### The Structure of the HbJAZ Genes in *H. brasiliensis*

Using MEME software, 12 conserved motifs were identified among the 18 HbJAZs (Figure 1A and Supplementary Figure S1). Prediction by the InterProscan database annotated motif 1 as the Jas domain, motifs 2 and 3 as ZIM domains, and motifs 4 and 5 as EAR domains (Yang et al., 2018). Motifs 6, 7, 8, 9, 10, 11, 12 were not annotated in the database. All the HbJAZs comprised motifs 1, 2, and 3. Four of the HbJAZs (HbJAZ3.0/ 4.0/9.0a/9.0b) contained eight motifs (1, 2, 3, 4, 5, 6, 7, 8). Four HbJAZs (HbJAZ5.0/6.0/1.0/2.0) included motif 10; three of them (HbJAZ5.0/6.0/1.0) owned extra motif 9. Motif 11 existed in eight HbJAZs (HbJAZ1.0/2.0/6.0/10.0a/10.0b/11.0/12.0/13.0) while motif 12 was found in other three HbJAZs (HbJAZ8.0b/8.0c/8.0d).

Exon–intron organization analysis of the 18 *HbJAZs* uncovered that most of the *HbJAZs* had at least two introns, except for three genes (*HbJAZ8.0b/8.0c*/*8.0d*) which had no intron (Figure 1B). Both the ZIM and Jas domains were separated by several introns in nine genes (*HbJAZ3.0*/ *4.0*/*9.0a*/*9.0b*/*10.0a*/*10.0b*/*11.0*/*12.0*/*13.0*). The ZIM domains of *HbJAZ7.0* and *HbJAZ8.0a* and the Jas domains of *HbJAZ1.0*, *HbJAZ2.0*, and *HbJAZ6.0* were however separated by one intron.

**FIGURE 2:**
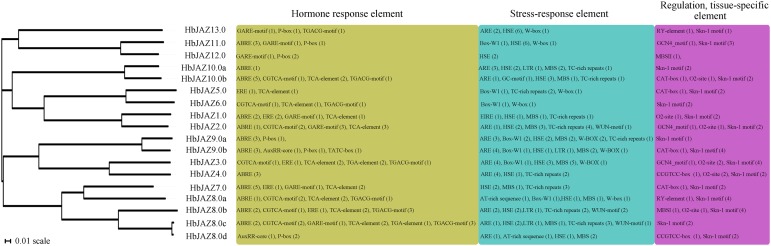
*Cis*-elements analysis of *HbJAZ* gene promoters. 1,500 bp genomic sequences upstream of the translational initiation codon of *HbJAZ* genes were analyzed by Plantcare. The yellow, blue, pink represented the elements related to hormones response, stresses response, tissues regulation, respectively. Number within the parentheses represented the number of *cis*-element.

Several *cis*-elements were identified in the 1,500 bp upstream sequence of the 18 *HbJAZs* (Figure 2). They were classified into three categories: hormone responsive, stress responsive, and developmental regulation. For the hormone responsive category, eight *HbJAZs* (*HbJAZ2.0/ 3.0/6.0/8.0a/8.0b/8.0c/10.0b*/*13*) had JA responsive elements (TGACG-motif, CGTCA-motif), and five *HbJAZs* (*HbJAZ1.0/ 3.0*/*5.0*/*7.0*/*8.0b*) contained EREs. Additionally, the abscisic acid responsive element (ABRE) was present in the promoter regions of 12 *HbJAZs* (*HbJAZ1.0*/ *2.0*/*4.0*/*5.0*/*7.0*/*8.0a*/*8.0b*/*8.0c*/*9.0a*/*9.0b*/*10.0a*/*10.0b*/*11.0*). Four *HbJAZs* (*HbJAZ3.0*/*8.0c*/*8.0d*/*9.0b*) contained auxin responsive elements (TGA-element, AuxRR-core), nine *HbJAZs* (*HbJAZ1.0*/*2.0*/*7.0*/*8.0c*/*8.0d*/*9.0a*/*9.0b*/*11.0*/*12.0*/*13.0)* had gibberellin responsive elements (GARE-motif, P-box), and eight *HbJAZs* contained salicylic acid responsive elements (*HbJAZ1.0*/*2.0*/*3.0*/*5.0*/*6.0*/*7.0*/*8.0a*/*8.0b*/*8.0c*/*10.0b*; TCA-element). For the stress responsive category, 11 *HbJAZs* (*HbJAZ2.0/3.0/5.0/6.0/8.0a/8.0b/8.0c/9.0a/9.0b/11.0/13.0*) had wound responsive elements (WUN-motif, W-box, Box-W1), 16 *HbJAZs* (*HbJAZ1.0/2.0/3.0/4.0/7.0/8.0a/8.0b/8.0c/8.0d/9.0a/ 9.0b/10.0a/10.0b/11.0/12.0/13.0*) contained HSEs, four *HbJAZs* (*HbJAZ8.0b/8.0c*/*9.0b/10.0a*) had LTRs, and 11 *HbJAZs* (*HbJAZ1. 0/2.0/3.0/7.0/8.0a/8.0c/8.0d/9.0a/9.0b/10.0a/10.0b*) contained drought response elements (MBS). For the developmental regulation category, six *HbJAZs* (*HbJAZ4.0/ 5.0/7.0/8.0d/9.0a/10.0b*) contained meristem specific elements (CAT-box, CCGTCC-box) and seven *HbJAZs* (*HbJAZ1.0/2.0/ 3.0/4.0/8.0b/10.0b/12.0*) had secondary metabolic biosynthesis regulation specific elements (MBSIs) (MBSI, MBSII, O2-site).

### Phylogenetic Analysis and Classification of the HbJAZ Proteins

**FIGURE 3:**
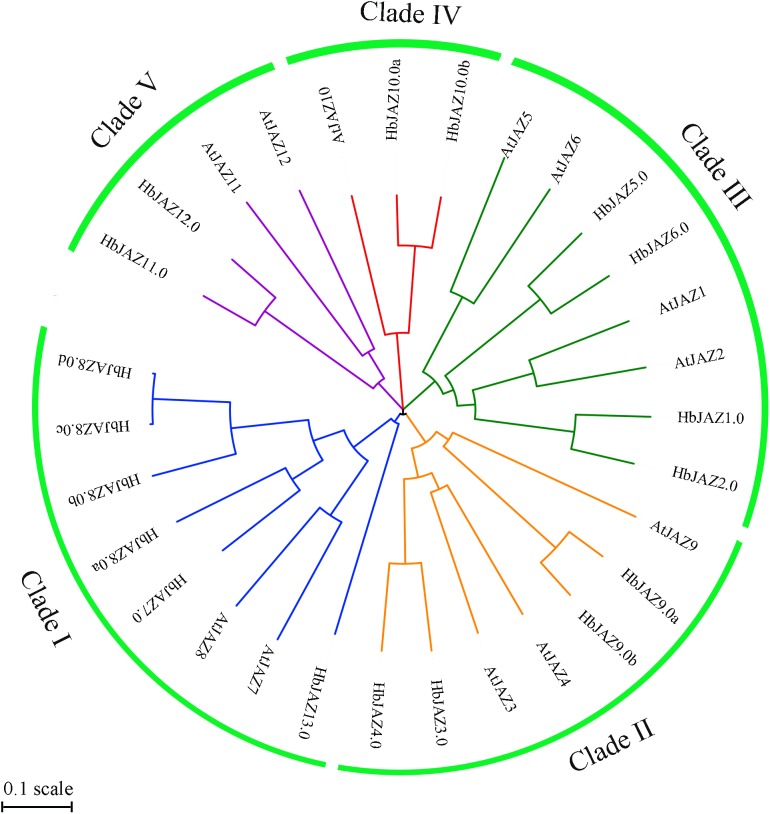
Phylogenetic tree of the HbJAZs and AtJAZs. The phylogenetic tree was calculated by Multiple Sequence Alignment software based on neighboring-joining phylogeny test, and displayed by iTOL software.

The 18 identified HbJAZs and 12 *Arabidopsis* JAZs (AtJAZs) were used to construct a phylogenetic tree (Figure 3). All the JAZs distinctly grouped into five clusters (I, II, III, IV, and V). Cluster I included two AtJAZs (AtJAZ7 and AtJAZ8) and six HbJAZs (HbJAZ7.0/8.0a/8.0b/8.0c/8.0d), Cluster II contained three AtJAZs (AtJAZ3/4/9) and four HbJAZs (HbJAZ3.0/4.0,/9.0a,/9.0b), Cluster III included four AtJAZs (AtJAZ1/2/5/6) and four HbJAZs (HbJAZ1.0/2.0/5.0/6.0), Cluster IV contained AtJAZ10, HbJAZ10.0a and HbJAZ10.0b, and Cluster V included two AtJAZs (AtJAZ11 and AtJAZ12) and two HbJAZs (HbJAZ11.0 and HbJAZ12.0).

### Interaction Among the HbJAZ Proteins

**FIGURE 4:**
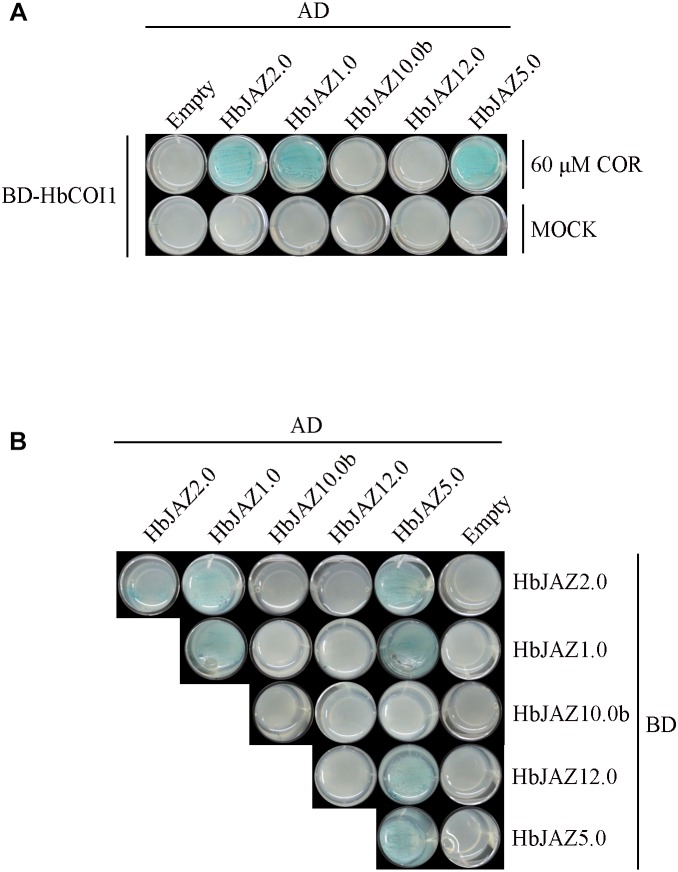
Interaction analysis of HbJAZ proteins. **(A)** Coronatine-dependent interaction between HbCOI1 and HbJAZs. **(B)** Homo- and hetero-meric interactions among HbJAZs. Controls for autoactivation were provided by transformation with the corresponding empty vector.

Five HbJAZs were selected for interaction analysis via yeast two-hybrid assay (Figure 4). The interactions among HbJAZs and HbCOI were COR-dependent (Figure 4A). In the presence of 60 μM COR, three HbJAZs (HbJAZ1.0/2.0/5.0) interacted with HbCOI1 (Figure 4A). These interactions also occurred in the presence of 20 μM COR (Supplementary Figure S2). The interactions among the HbJAZs were COR-independent. HbJAZ5.0 could respectively interact with HbJAZ1.0, HbJAZ2.0, HbJAZ5.0 and HbJAZ12.0, the HbJAZ1.0 interacted with itself and HbJAZ2.0, and HbJAZ2.0 interacted with itself (Figure 4B).

### The Expression Patterns of HbJAZ Genes in Vascular Cambium Region in Response to COR

**FIGURE 5:**
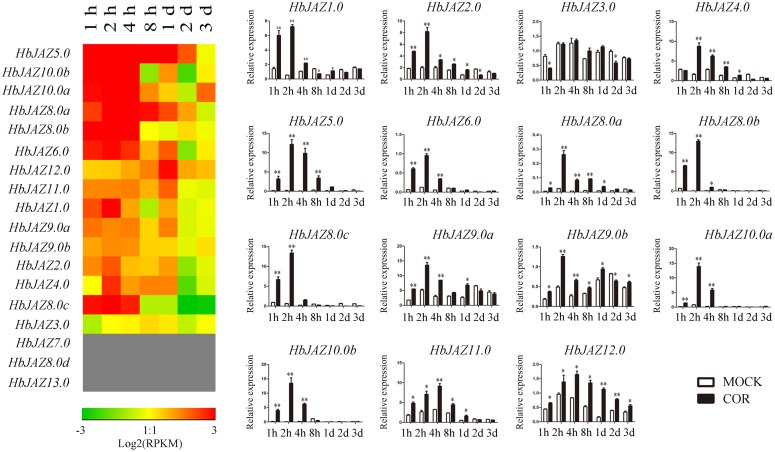
Expression profiles of *HbJAZs* in cambium upon COR treatment. The qRT-PCR results were displayed by heatmap **(left)** and bar chart **(right)**. Red showed high expression and green showed low expression. Bar represented the Log 2 value. ^∗^, ^∗∗^ represented 0.05, 0.01 significant difference.

Among the 18 *HbJAZs*, the transcripts of *HbJAZ7.0, HbJAZ8.0d*, and *HbJAZ13.0* were absent from the vascular cambium region (Figure 5). COR upregulated the 14 *HbJAZs* while had little influence on the expression of *HbJAZ3.0*. The duration of up-regulated expression was different among the 14 *HbJAZs*. The up-regulated expression lasted 2 h for *HbJAZ8.0c*, 4 h for six *HbJAZs* (*HbJAZ1.0/6.0/8.0b/9.0a/10.0a/10.0b*), 1 day for five *HbJAZs* (HbJAZ2.0/4.0/8.0a/9.0b/11.0) and 3 days for *HbJAZ12.0* (Figure 5). Except for HbJAZ11.0 and HbJAZ12.0, the expression level reached peak at 2 h for 12 of the 14 *HbJAZs.* The expression level of *HbJAZ11.0* and *HbJAZ12.0* reached the peak at 4 h. The transcript levels of seven *HbJAZs* (*HbJAZ1.0/5.0/8.0a/8.0b/8.0c/10.0a/10.0b*) were more than 10 times of the control at 2 h (Figure 5).

### The Expression Patterns of HbJAZ Genes in the Latex in Response to Tapping and Ethrel Treatment

**FIGURE 6:**
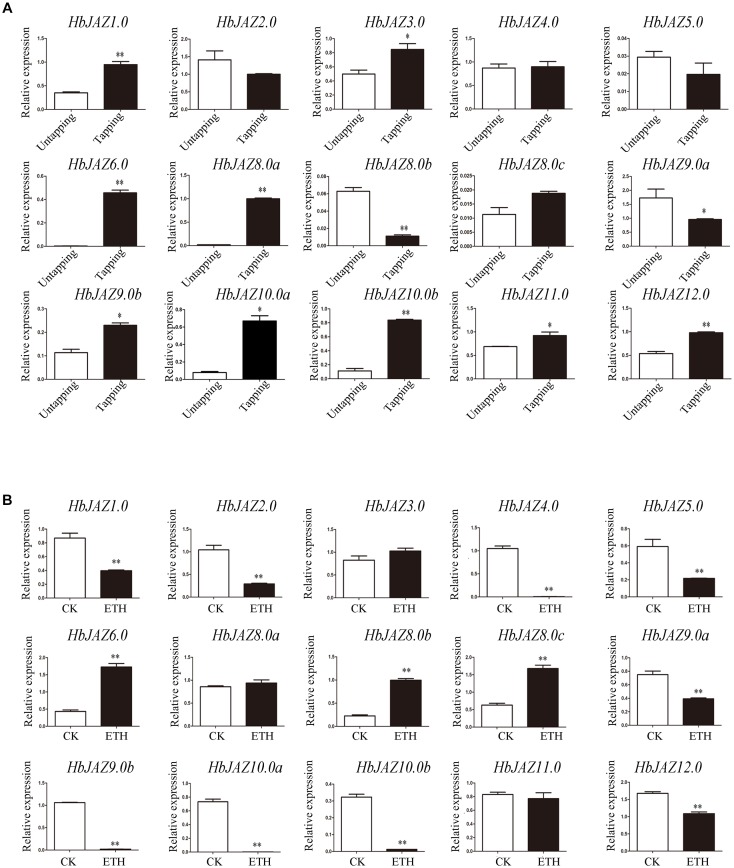
Expression profiles of *HbJAZs* in latex upon tapping **(A)** or ethrel treatment **(B)**. Bar represented the Log 2 value of qRT-PCR results. ^∗^, ^∗∗^ represented 0.05, 0.01 significant difference.

The transcripts of three genes (*HbJAZ7.0*, *HbJAZ8.0d*, and *HbJAZ13.0*) which were absent from the vascular cambium region, were also absent from the latex. Of the 14 COR-upregulated *HbJAZs* in the vascular cambium region, eight genes (*HbJAZ1.0/6.0/8.0a/9.0b/10.0a/10.0b/11.0/12.0*) in the latex were up-regulated while two genes (*HbJAZ8.0b* and *HbJAZ9.0a*) were down-regulated by tapping. The other four genes (*HbJAZ2.0/4.0/5.0/8.0c*) had little response to tapping (Figure 6A). By contrast, nine genes (*HbJAZ1.0/2.0/4.0/5.0/9.0a/9.0b/10.0a/10.0b/12.0*) were down-regulated while three genes (*HbJAZ6.0*, *HbJAZ8.0b*, and *HbJAZ8.0c*) were up-regulated by ethrel treatment. The other two genes (*HbJAZ8.0a* and *HbJAZ11.0*) had little response to ethrel treatment (Figure 6B). Tapping also up-regulated the expression of *HbJAZ3.0* which had little response to COR and ethrel treatment (Figure 6). Except for HbJAZ6.0 and HbJAZ9.0a, the expression patterns of 13 of the 15 *HbJAZs* which transcripts were present in the latex were different between tapping and ethrel treatment (Figure 6). The *HbJAZ6.0* was up-regulated and *HbJAZ9.0a* was down-regulated by either tapping or ethrel treatment (Figure 6).

## Discussion

In the past decade, JAZ family has been widely identified in different plant species. There are 12 JAZs in *Arabidopsis* (Thines et al., 2007), 15 JAZs in rice (Ye et al., 2009), 18 JAZs in apple (Li et al., 2015), 30 JAZs in cotton (Li et al., 2017), 13 JAZs in tomato (Chini et al., 2017), and 11 JAZs in grape (Zhang et al., 2012). The following features could distinguish JAZs from other TIFY family proteins: (1) the C-terminal located Jas domain at, (2) the jasmonate-dependent interaction between JAZ and COI1, and (3) the formation of homo- and hetero-dimers within the JAZ subfamily. Several versions of the rubber tree genome have published in the past few years (Rahman et al., 2013; Lau et al., 2016; Tang et al., 2016; Pootakham et al., 2017; Makita et al., 2018). As Tang et al. (2016) version has less scaffolds number (7,453) and longer scaffolds N50 (1.28 Mb) (Supplementary Table S3), the genome-wide identification of JAZs was based on this version in the present study. As a result, 18 *HbJAZs* were identified in rubber tree. Ten of them (*HbJAZ2.0/3.0/6.0/8.0a/8.0c/9.0a/9.0b/10.0a*/*11.0*/*12.0*) are identical to those that have ever been reported (Tian et al., 2010; Pirrello et al., 2014; Hong et al., 2015). The other eight *HbJAZs* (*HbJAZ1.0/4.0/5.0/7.0/8.0b/8.0d/10.0b*/*13.0*) are novel (Table 1). In addition to the bioinformatic identification, evidence are provided for the interaction between some members and their interaction with HbCOI1 in the presence of COR (Figure 4).

Gene family expansion is a source of new gene generation (Jia et al., 2015). Tandem duplication and segmental duplication are the two strategies for expanding the number of genes in a family (Cannon et al., 2004; Jin et al., 2017). Compared to genes with tandem duplication, genes that come from segmental duplication at different chromosomes or scaffolds have been suggested to perform different functions (Dennis and Eichler, 2016). In rice, the clusters of *OsJAZ9/10/11* and *OsJAZ12/13/14* are from tandem duplication, while the clusters of *OsJAZ3/4*, *OsJAZ2/15*, and *OsJAZ6/7* are from segmental duplication (Ye et al., 2009). In apple, Li et al. (2015) determine that the clusters of *MdJAZ3/4/5/6/7/8*, *MdJAZ11/12*, and *MdJAZ13/14* are from tandem duplication, while the clusters of *MdJAZ4/7*, *MdJAZ7/3*, *MdJAZ1/15*, *MdJAZ12/18*, and *MdJAZ18/11* are from segmental duplication. In grape, Zhang et al. (2012) report that VvJAZ5/6/7/8 are from tandem duplication, while the pairs of *VvJAZ1/11* and *VvJAZ4/9* are from segmental duplication. In rubber tree, the lack of high-density genetic map precludes the identification of paralogs derived from Whole Genome Duplication (WGD) or segmental duplication. However, Zou et al. (2016, 2017, 2019) identify several segmental duplication events in rubber tree genome, and firstly reveal that ρ WGD occurred in rubber tree is shared by cassava. In the present study, we identified at least three clusters (*HbJAZ3.0/4.0*, *HbJAZ9.0a/9.0b*, *HbJAZ10.0a/10.0b*) that were segmental duplications while one pair (*HbJAZ8.0c/8.0d*) was tandem duplicated, based on the scaffold location, gene structure, and protein conserved motifs (Figure 1 and Table 1). It is clear that the two segmentally duplicated clusters (*HbJAZ3.0/4.0*, *HbJAZ9.0a/9.0b*) have differential expression patterns in response to COR, ethrel or tapping treatments, suggesting the putative functional diversity of these genes. Phylogenetic tree analysis of JAZs from multiple species shows that the JAZ members that come from segmental duplication separate into different clusters while those from the tandem duplication are clustered together. The exception to this is JAZ members in rice (Supplementary Figure S3 and Supplementary Table S4), which may be attributed to the fact that rice is a monocot while the other species are dicots.

It has been suggested that JA signaling is involved in the regulation of plant development (Singh et al., 2015; Huang et al., 2017). In *Arabidopsis*, AtJAZ7 and AtJAZ10 are the key regulators in JA-mediated cambium differentiation (Sehr et al., 2010). Li et al. (2015) show knockdown of *AsJAZ1* decreases the number of nodules in *Astragalus sinicus*. In cotton, over-expression of *GhJAZ2* inhibits both lint and fuzz fiber initiation and reduces the fiber length (Hu et al., 2016). Over-expression of *SlJAZ2* in tomato directly activates meristem-related genes and results in production of more leaves and flowers (Yu et al., 2018). To further explore the key members involved in secondary laticifer differentiation of rubber trees, we constructed a phylogenetic tree using the 18 HbJAZs together with AtJAZ7, AtJAZ10, AsJAZ1, GhJAZ2, SlJAZ2 (Supplementary Figure S4 and Supplementary Table S4). It is intriguing that 11 of the HbJAZs (HbJAZ1.0/2.0/5.0/6.0/7.0/8.0a/8.0b/8.0c/8.0d/10.0a/10.0b) are homologous to the plant development-related homologs, and five of them (*HbJAZ5.0/8.0b/8.0c/10.0a/10.0b*) are significantly up-regulated greater than 10 times upon COR treatment. Moreover, a meristem specific element (CAT-box) is present in the promoter regions of *HbJAZ5.0* and *HbJAZ10.0b*. The CAT-box is a *cis-*regulatory element related to meristem expression. In *Brassica juncea*, mutation of the CAT-box in the promoter of *BjuA07.CLV1* disturbs the CLAVATA (CLV)/WUSCHEL (WUS) signaling pathway, leading to the enlargement of the shoot and floral meristem, and ultimately multilocular siliques (Xiao et al., 2018). Thus, we speculate that *HbJAZ5.0* and *HbJAZ10.0b* may play a key role in laticifer differentiation in *H. brasiliensis*.

Jasmonate is widely involved in the regulation of secondary metabolism in plants. Boter et al. (2015) show that degradation of JAZ3 activates a subset of JA-regulated genes in leaves, leading to anthocyanin accumulation in *Arabidopsis*. Mutation of the Jas motif of NtJAZ1 or NtJAZ3 reduces nicotine content in tobacco BY-2 cells (Shoji et al., 2008). In *Salvia miltiorrhiza*, over-expression of *SmJAZ3* or *SmJAZ8* notably reduces tanshinone production (Shi et al., 2016; Pei et al., 2018). Natural rubber is a typical secondary metabolite and its biosynthesis is significantly activated by tapping (Deng et al., 2018). The tapping-enhanced rubber biosynthesis is closely related to the activation of jasmonate signaling in laticifer cells (Deng et al., 2018). Exogenous methyl jasmonate enhances rubber biosynthesis while ethrel, inhibits rubber biosynthesis (Deng et al., 2018). In the present study, the expression patterns of *HbJAZs* in response to tapping were similar to those in response to COR, but generally opposite to the patterns in response to ethrel treatment (Figure 5, 6). The differences in the expression levels of 13 of the *HbJAZs* between the tapping and ethrel treatments may be associated with the different effects of methyl jasmonate and ethrel on rubber biosynthesis. Phylogenetic tree analysis reveals that 11 HbJAZs (HbJAZ1.0/2.0/3.0/4.0/7.0/8.0a/8.0b/8.0c/8.0d/9.0a/9.0b) are homologous to AtJAZ3, NtJAZ1, NtJAZ3, SmJAZ3, and SmJAZ8 (Supplementary Figure S4 and Supplementary Table S4), and tapping causes the expression level of *HbJAZ8.0a* and *HbJAZ8.0b* more than five times of control (Figure 6A). We also identified a secondary MBSI in the promoter region of *HbJAZ8.0b*. MBSI is present in the promoter of flavonoid biosynthetic genes, such as *chsJ*. In *Petunia hybrida*, the petal epidermis-specific MYB.Ph3 binds the MBSI element that regulates the biosynthesis of flavonoids (Solano et al., 1995). Based on the evidence described above, *HbJAZ8.0b* is likely a key regulator of natural rubber biosynthesis.

## Conclusion

Jasmonate signaling plays a vital role in the regulation of secondary laticifer differentiation and natural rubber biosynthesis. In the present study, JAZs, the repressors of jasmonate signaling, are genome-wide identified. Based on the computational analyses and gene expression patterns, *HbJAZ5.0* and *HbJAZ10.0b* might be key regulators of laticifer differentiation whereas *HbJAZ8.0b* was crucial for regulation of natural rubber biosynthesis in *H. brasiliensis*. The genome-wide identification of *HbJAZs* will facilitate the jasmonate signaling-mediated laticifer differentiation and natural rubber biosynthesis in rubber tree.

## Author Contributions

JC designed and carried out the experiment of this study and wrote the manuscript. YZ, JJ, SW, XM, and YC participated and analyzed data in the experiment. W-MT planned the study and participated in the design of the experiment. All authors have read and approved the manuscript in its final form.

## Conflict of Interest Statement

The authors declare that the research was conducted in the absence of any commercial or financial relationships that could be construed as a potential conflict of interest.

## Footnotes

1 https://www.ncbi.nlm.nih.gov/genome/?term=rubber%20tree%20genome

2 http://www.arabidopsis.org/

3 http://hmmer.org/

4 http://www.ncbi.nlm.nih.gov/cdd/

5 https://www.ebi.ac.uk/Tools/msa/

6 http://itol.embl.de/

7 http://expasy.org/

8 http://gsds.cbi.pku.edu.cn/

9 http://meme-suite.org/tools/meme

10 http://www.ebi.ac.uk/Tools/pfa/iprscan/

11 http://bioinformatics.psb.ugent.be/webtools/plantcare/html/

## Supplementary Material

FIGURE S1 Conserved amino acid sequences of 12 motifs.

FIGURE S2 Interactions between HbCOI1 and three HbJAZs in the presence of 20 μM COR.

FIGURE S3 Phylogenetic relationships of HbJAZs with other species homolog. The trees were constructed by neighboring-joining phylogeny test, and 1,000 bootstrap replicates. The accession numbers for the genes are provided in Supplementary Table S4. Red color represented tandem duplication, while blue color represented segmental duplication.

FIGURE S4 Phylogenetic relationships of HbJAZs with other species homolog related to plant development or secondary metabolites biosynthesis. The trees were constructed by neighboring-joining phylogeny test, and 1,000 bootstrap replicates. The accession numbers for the genes are provided in Supplementary Table S4. Blue circle represented pigments biosynthesis related TFs in other species. Red circle represented genes identified in this study.

TABLE S1 Primers used in this paper.

TABLE S2 The full amino acid sequences of the 18 HbJAZs.

TABLE S3 Evaluation of four rubber tree genome versions.

TABLE S4 Gene accession numbers used in this paper.

## Abbreviations

CDSs: coding DNA sequences
COI1: CORONATINE INSENSITIVE1
COR: coronatine
EAR: ERF-associated amphiphilic repression
EREs: ethylene responsive elements
HMM: hidden Markov model
HSEs: heat responsive elements
JAZ: jasmonate ZIM-domain
LTR: low-temperature response elements
qRT-PCR: quantitative real-time polymerase chain reaction.
