# Supplementary figures and images for: Genome-Wide Identification and Characterization of the JAZ Gene Family in Rubber Tree (Hevea brasiliensis)
Source: Front Genet. 2019 May 1;10:372. doi: 10.3389/fgene.2019.00372 (PMC6504806; doi:10.3389/fgene.2019.00372)

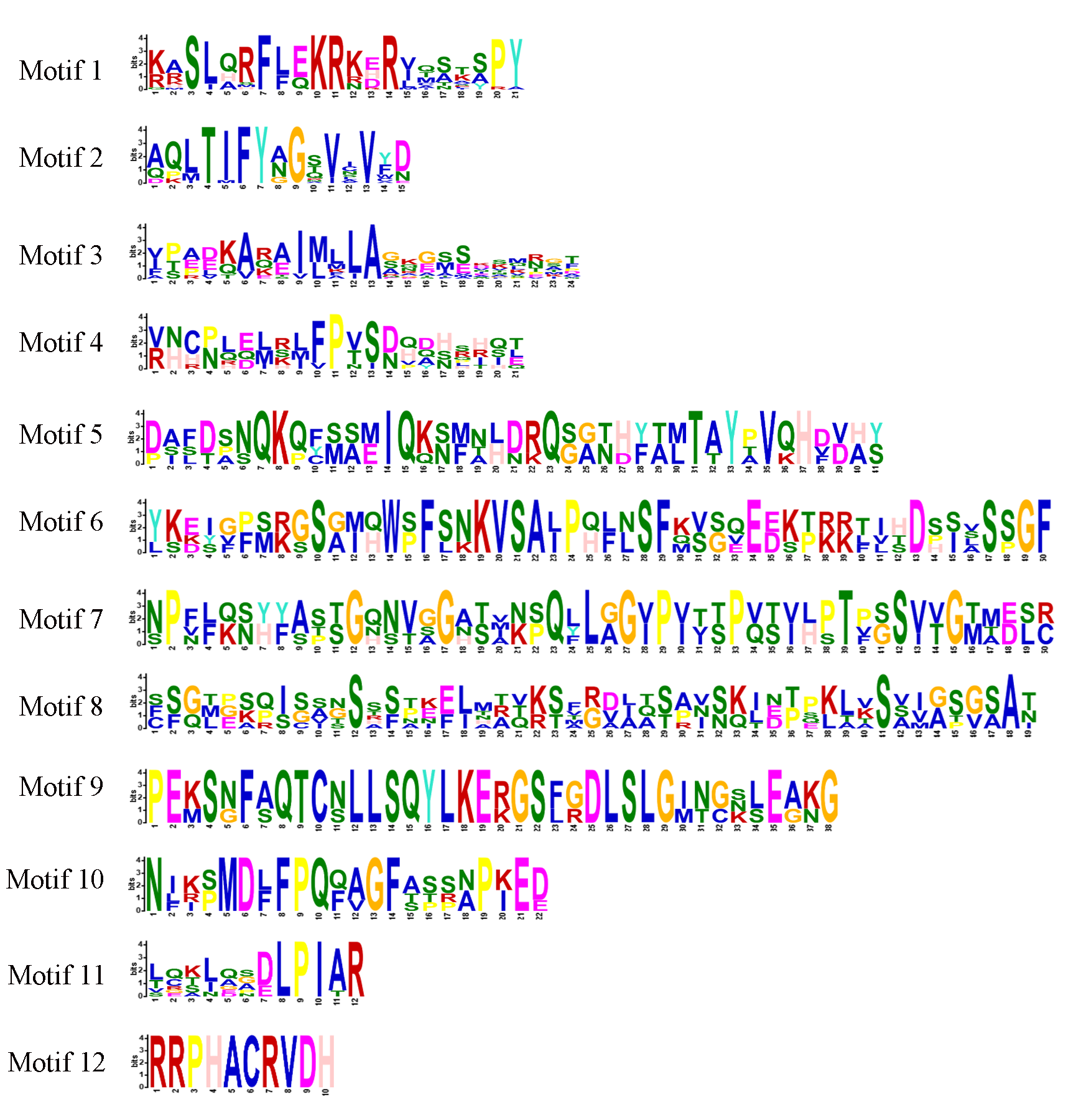

Supplement: FIGURE S1 — Conserved amino acid sequences of 12 motifs. [file Image_1.TIF]

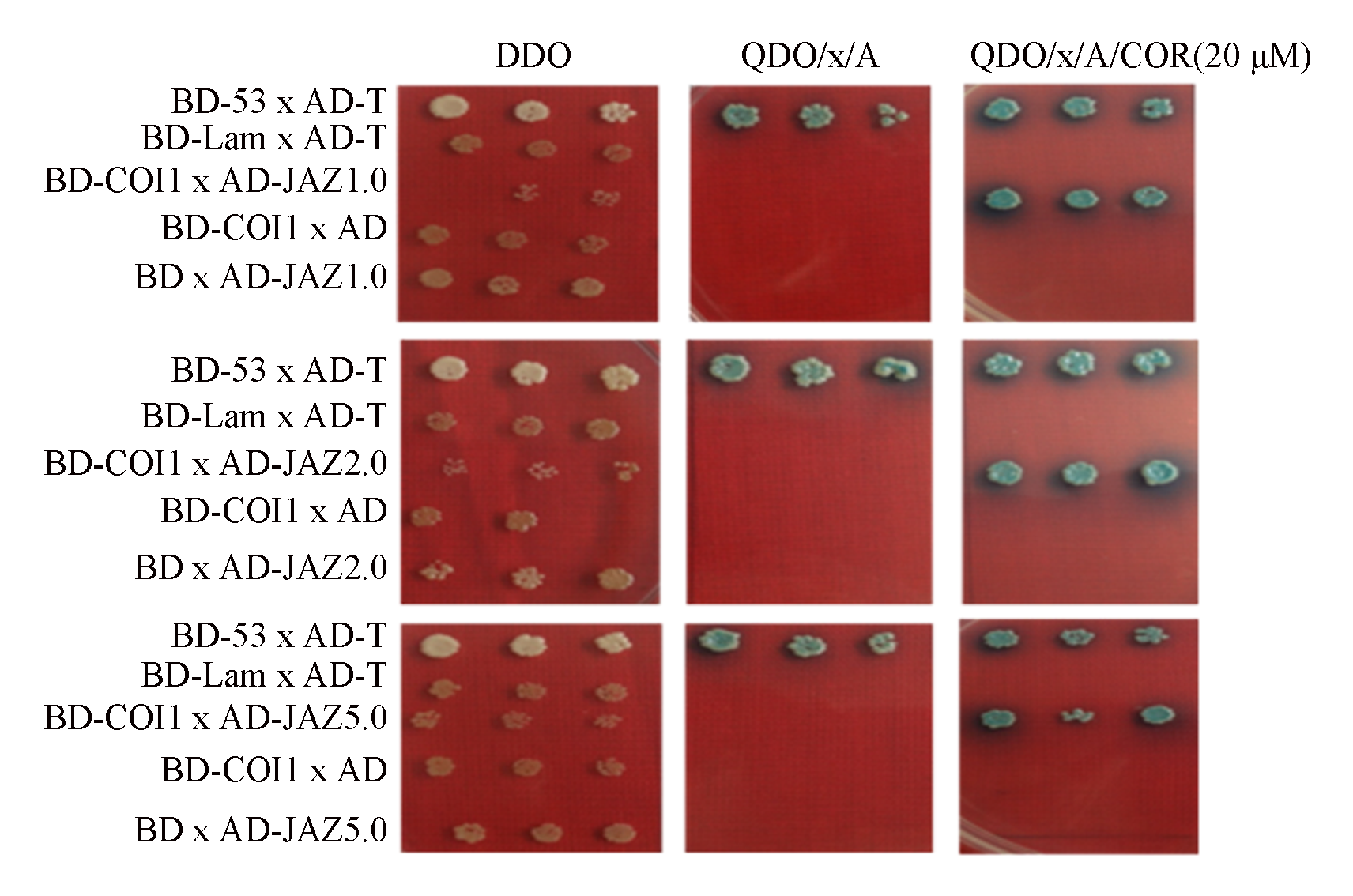

Supplement: FIGURE S2 — Interactions between HbCOI1 and three HbJAZs in the presence of 20 μM COR. [file Image_2.TIF]

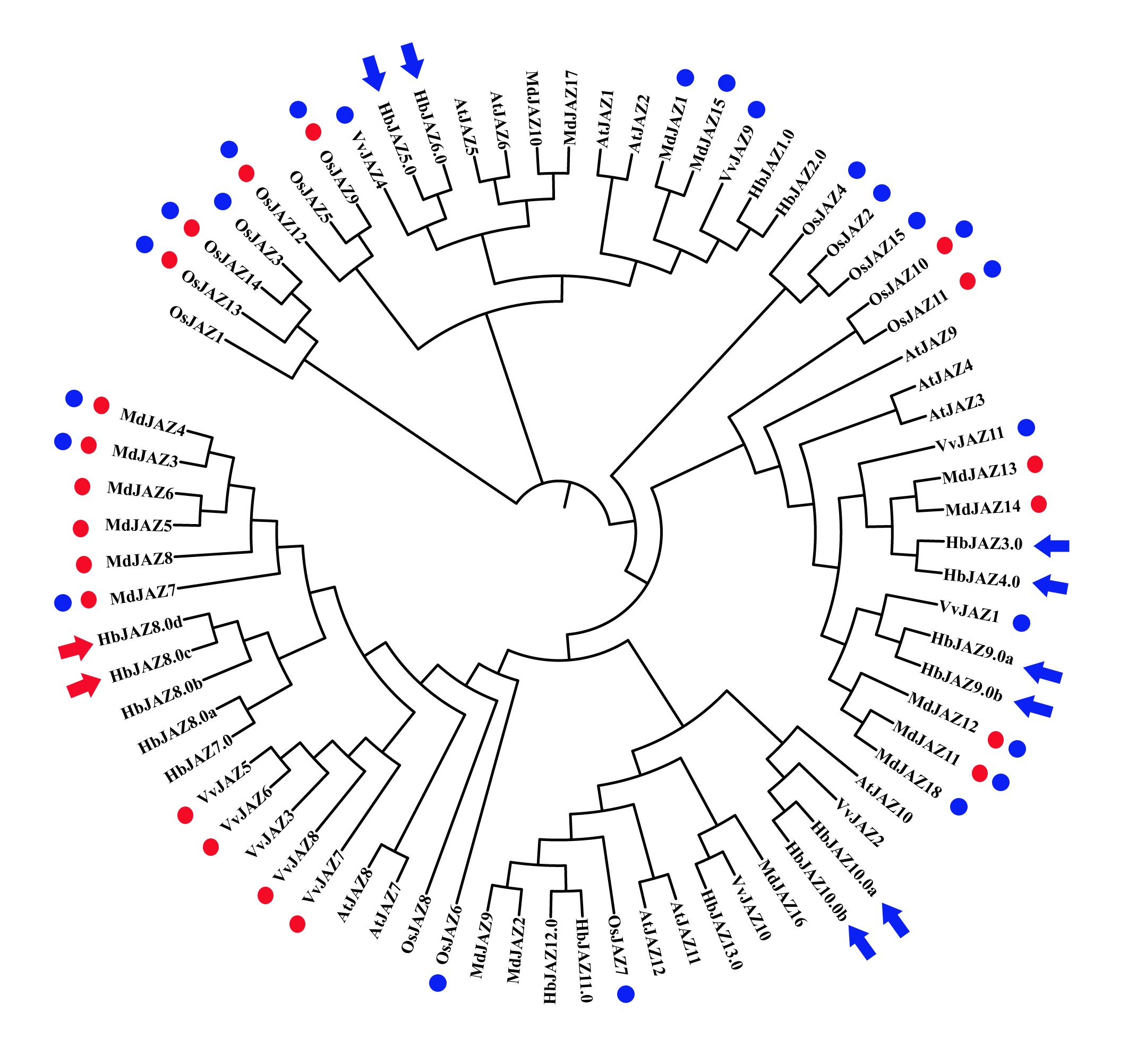

Supplement: FIGURE S3 — Phylogenetic relationships of HbJAZs with other species homolog. The trees were constructed by neighboring-joining phylogeny test, and 1,000 bootstrap replicates. The accession numbers for the genes are provided in Supplementary Table S4. Red color represented tandem duplication, while blue color represented segmental duplication. [file Image_3.TIF]

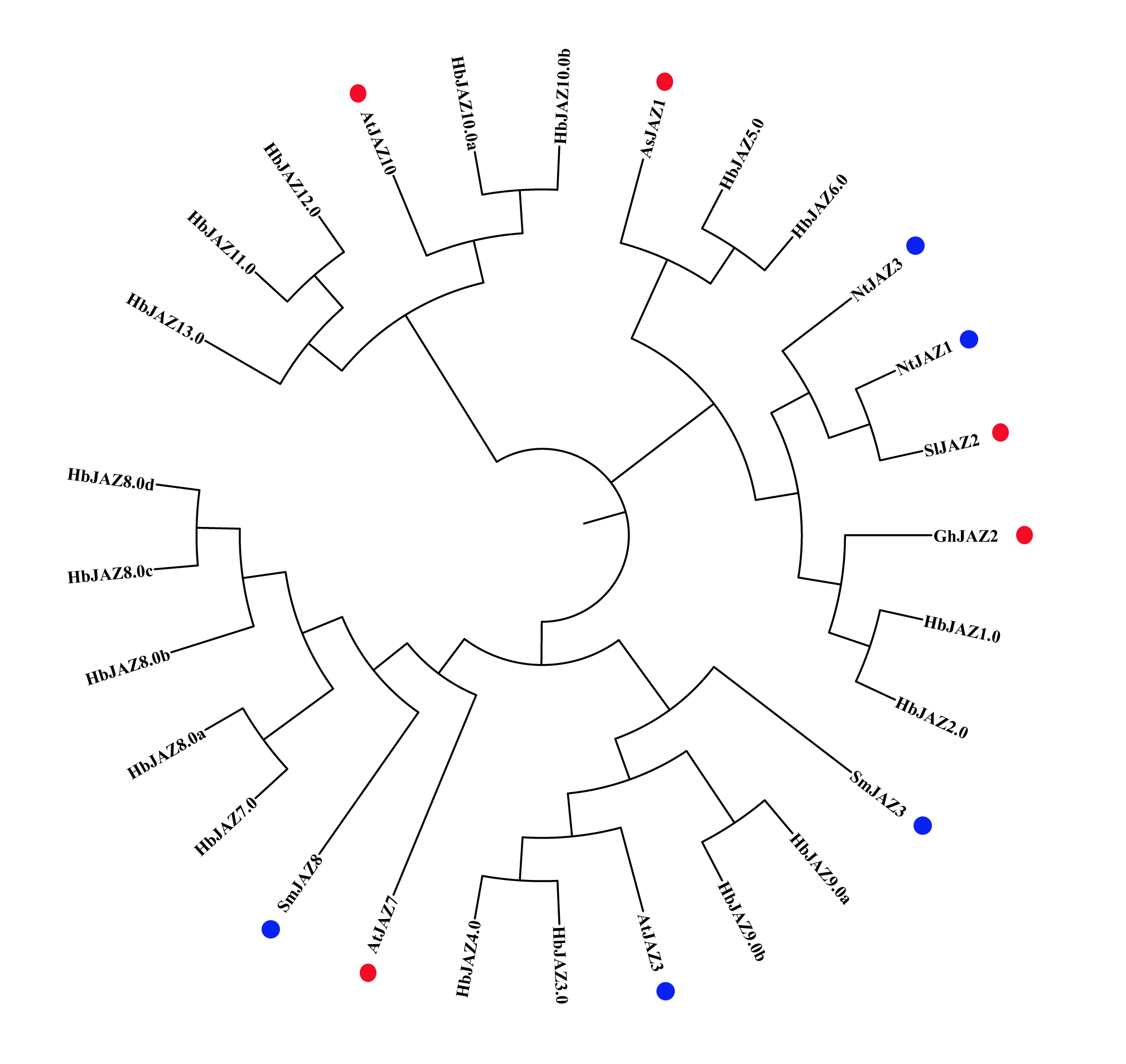

Supplement: FIGURE S4 — Phylogenetic relationships of HbJAZs with other species homolog related to plant development or secondary metabolites biosynthesis. The trees were constructed by neighboring-joining phylogeny test, and 1,000 bootstrap replicates. The accession numbers for the genes are provided in Supplementary Table S4. Blue circle represented pigments biosynthesis related TFs in other species. Red circle represented genes identified in this study. [file Image_4.TIF]
