# Supplementary material for: Genome-Wide Identification and Characterization of the JAZ Gene Family in Rubber Tree (Hevea brasiliensis)
Source: Front Genet. 2019 May 1;10:372. doi: 10.3389/fgene.2019.00372 (PMC6504806; doi:10.3389/fgene.2019.00372)
Supplement: TABLE S1 — Primers used in this paper. [file Table_1.DOCX]

**Additional file 1**

**Table S1. Primers used in this paper.**

| Primers Name | Primer sequence |
| --- | --- |
| Primers for qRT-PCR |  |
| HbJAZ1.0-qF | CTCCTTCTAAGCCAGC |
| HbJAZ1.0-qR | GGATTCAACATCCGCC |
| HbJAZ2.0-qF | TCCATACCAAACAAGT |
| HbJAZ2.0-qR | CCCGCTACAAAAATAC |
| HbJAZ3.0-qF | CAAGCCCTATTTCTGTGAACTCT |
| HbJAZ3.0-qR | GAACCAGGTGCTACTGAACTAACT |
| HbJAZ4.0-qF | CTCACACGAGAAAACG |
| HbJAZ4.0-qR | TGTTCCTTTGACCCCA |
| HbJAZ5.0-qF | CACTTGATTGTTGACC |
| HbJAZ5.0-qR | ATTATCGTACGTTGCT |
| HbJAZ6.0-qF | ATGGCGAACTTGGTCCAGAA |
| HbJAZ6.0-qR | TTGTTGTTGGTGGCCTAGAT |
| HbJAZ7.0-qF | ACAACTTCTCCTTACA |
| HbJAZ7.0-qR | AGCCACACTGCTCATT |
| HbJAZ8.0a-qF | ATGAGACGAAACTGCAATCT |
| HbJAZ8.0a-qR | GCTCTTGCCTGGAGCTCCGT |
| HbJAZ8.0b-qF | TTCAAACAACTTACCC |
| HbJAZ8.0b-qR | AAAATTTACAAGCGTC |
| HbJAZ8.0c-qF | CGTCTTCGTTGTCGTCGGA |
| HbJAZ8.0c-qR | TCAATGGTGATCGACCCTGCA |
| HbJAZ8.0d-qF | AACCTGTATCGCCAAC |
| HbJAZ8.0d-qR | AAAAATGAAATCCCTT |
| HbJAZ9.0a-qF | ATGGAGAGAGATTTTATGGGT |
| HbJAZ9.0a-qR | ATCTTCTTGAGAAACCATGA |
| HbJAZ9.0b-qF | GCTCGGTTTTTGGAGA |
| HbJAZ9.0b-qR | AGAGAACAGGTCGCCT |
| HbJAZ10.0a-qF | ATGTCGAGAGCAACTGTCGA |
| HbJAZ10.0a-qR | GAGTACTTTGCTGATTGGCA |
| HbJAZ10.0b-qF | TTAAGGATAGGGACCT |
| HbJAZ10.0b-qR | ACCCATTACTAAAACG |
| HbJAZ11.0-qF | TTACTGCCAACCTTGC |
| HbJAZ11.0-qR | TGTTCCTTTGACCCCA |
| HbJAZ12.0-qF | ATGGAGGGTGATTCTGATTC |
| HbJAZ12.0-qR | GATCTGAAGTAGGGACAGT |
| HbJAZ13.0-qF | AAGCCAAGAGCAGATG |
| HbJAZ13.0-qR | AGCAATAAATACAAAA |
| HbUBC2b-qF | CGACCAAGTTTTCATTTCGGGTG |
| HbUBC2b-qR | AGTCTCTTCTTTGCTGGGGTTG |
| Primers for protein interaction |  |
| HbJAZ1.0-AD-F | GGAGGCCAGTGAATTCATGGCTGGTTCGCCCGATTT |
| HbJAZ1.0-AD-R | CGAGCTCGATGGATCCTCAAATATGATTAGCAATCG |
| HbJAZ2.0-AD-F | GGAGGCCAGTGAATTCATGAATTTGTTTCCAATGAG |
| HbJAZ2.0-AD-R | CGAGCTCGATGGATCCCTACTGCAAAGATTGACCAG |
| HbJAZ5.0-AD-F | GGAGGCCAGTGAATTCATGGCAAACTTGGTTCACAA |
| HbJAZ5.0-AD-R | CGAGCTCGATGGATCCCTATAACTTAAGCTCAAGCT |
| HbJAZ10.0b-AD-F | GGAGGCCAGTGAATTCATGTCTAGAGCAAGTGTCGA |
| HbJAZ10.0b-AD-R | CGAGCTCGATGGATCCCTATAAACGACAAACAGATT |
| HbJAZ12.0-AD-F | GGAGGCCAGTGAATTCATGGGATCTTGCAAGGAAGG |
| HbJAZ12.0-AD-R | CGAGCTCGATGGATCCTTAAGCAAGGTTGGCAGCAA |
| HbJAZ1.0-BD-F | CATGGAGGCCGAATTCATGGCTGGTTCGCCCGATTT |
| HbJAZ1.0-BD-R | GCAGGTCGACGGATCCTCAAATATGATTAGCAATCG |
| HbJAZ2.0-BD-F | CATGGAGGCCGAATTCATGAATTTGTTTCCAATGAG |
| HbJAZ2.0-BD-R | GCAGGTCGACGGATCCCTACTGCAAAGATTGACCAG |
| HbJAZ5.0-BD-F | CATGGAGGCCGAATTCATGGCAAACTTGGTTCACAA |
| HbJAZ5.0-BD-R | GCAGGTCGACGGATCCCTATAACTTAAGCTCAAGCT |
| HbJAZ10.0b-BD-F | CATGGAGGCCGAATTCATGTCTAGAGCAAGTGTCGA |
| HbJAZ10.0b-BD-R | GCAGGTCGACGGATCCCTATAAACGACAAACAGATT |
| HbJAZ12.0-BD-F | CATGGAGGCCGAATTCATGGGATCTTGCAAGGAAGG |
| HbJAZ12.0-BD-R | GCAGGTCGACGGATCCTTAAGCAAGGTTGGCAGCAA |
| HbCOI1-BD-F | CATGGAGGCCGAATTCATGGAAGAGGAGAATCAGAG |
| HbCOI1-BD-R | GCAGGTCGACGGATCCTCATTTACAGCTCTCTATGCGG |
