# Supplementary material for: Genome-Wide Identification and Characterization of the JAZ Gene Family in Rubber Tree (Hevea brasiliensis)
Source: Front Genet. 2019 May 1;10:372. doi: 10.3389/fgene.2019.00372 (PMC6504806; doi:10.3389/fgene.2019.00372)
Supplement: TABLE S2 — The full amino acid sequences of the 18 HbJAZs. [file Table_2.DOCX]

**Additional file 2**

**Table S2. The full amino acid sequences of the eighteen HbJAZs.**

**HbJAZ1.0**

MAGSPDFVEFAGQKGARWPEMSGFSQTCSLLSQYLKEKGSLGDLSLGMTCSSEGNGTPEMVRQVATTMNLFPMSEKQGDVSTTNKPTTRTNLISMDLFPQQAGFTTSAPKEDVQKSRDSRINKAATPEPQTAQMTIFYAGQVIVFNDFPADKAKEVMLLAGKGSSKGLTGFLPSVPVKNHPVFAPNVAKTPVESSGSIPPSSNAVPNFGNNLNQERMQPPTQSIANDLPIARKASLHRFLEKRKDRITARSPYQTIGLSGSPSKPAESKWLLNLRSNTIANHI*

**HbJAZ2.0**

MNLFPMSEKQVDVSSRNMATPPRTNFRSMDLFPQQAGFSPSAPKEDVQKSLDSSVNKAATPEPQTAPMTIFYAGQVIVFNDFPADKVKEVMLLASKGSSQSLTGFPSVPVKSHPVFDPNVAKAPVESTSSIPPNSNPVPSFGNNLNQERVQSPSQTIASDLPIARRASLHRFLEKRKDRITASARAPYQTSRGLSASPSKPAESKPWLGLAGQSLQ*

**HbJAZ3.0**

MFSTRCQFPIWFLVLRHLNNLSKYFPMRGSAMQWSFLKKVSAIPQFLSFKSGEEESPRKTIHDPIASSGFTPISTADALDSNQKQYSSMIQKNMALDRQGANHYAMTAYAVQHVDAYPVHRPQQMRIFPVINHQNPTITVSMSNPNLQSHFASTGNNVGGNSINSQYLAGVPIVSPVSVHPTPSSVVGTTDLRNGSKSSGAPAQLTIFYAGSVCVYEDISPEKAQAIMLLAGHGSSVTQNKAISPVQVRTPIPRPSADDGFVGSKIHTASPCSGLPSPISVTSSSAIELTTVKSVGALASANNQIETSKTVSSVAPGSAIPIPAGAVPQARKASLARFLEKRKERVMNTSPYNVSKKSLDCSATECDDVSLSINSLSSPGHQ*

**HbJAZ4.0**

MERDFLGLGSKNVPVTVKKELTDGYKDSVPMRGSAMQWSFSNKVSAIPQFLSFQSGVEEKPRKTIHDHISSSGFTPISTASQKPCSSMIQQNMTLDKQGANHYAMTTYTVKHFDAYPVHCPQQIRIFPVSDVQNQRITASMSSPVLQSHFSSTGHNVGGHSMNSQFLAGVPIISPVSVHSTPSSIVGTADLRNGSKSSGAPAQLTIFYAGSVFIYDDISPEKAQAIMLLAGNGSSVTQNKTVSPAQVQAPIRRPSAGDDLVGNKIHSASPCSGLPSPISISSSSNNELATVKSMRDLASRNNQTEPPKAVSSVGTGSATLIPAVAVPQARKASLARFLEKRKERMMNTSPYNVSKKSPDSSSAEYDGVSLAIN*

**HbJAZ5.0**

MANLVHKSGKAASPEKSNFAQTCNLLSQYLKERGSFGDLSLGINGNLEAKGPEASRPPATTLNLLSNIENSAETSRKNSVPAPNIKPMDFFPQFAGFASRNPIEDSTAKPADLRKSSRAVDPGTAQMTIFYAGQVIVYDDFPADKAKEIMALASKGISNAQTGFTASTSAMYKVNPTIAIASNNAPEGLRLHPQVNGSDMPIVGKASLHRFFEKRKERVASKAPYQLNNPSSLARPRPSKESNHLIVDLEAQSSKQLELKL*

**HbJAZ6.0**

MANLVQNKSSGKAAPEKSNFAQTCNLLSQYLKERGSFRDLSLGINGKLEAKGPEASRPPTTTLNLLSNIEISAETSRQNSVPSANIKPMDFFPQFVGFASPNPIEEDSIANKPADLRKSSREEPGTAQLTIFYAGQVIVYDDFPADKAKEIMALASKGTSNSKNGFTTTASTSAMDKTNSIASNNNAREGLRLQTQANGSDLPIARRASLHRFFEKRKDRVASKAPYQLNNPSSPARPRPDEESNPIIIDLEVEGQSSKQLELKL*v

**HbJAZ7.0**

MRRNCNLELHLVPNSDPDHLHHQPILEEKESSSDEQRQQLTIFYNGNVCVCNVTELQARAILLLARGEMEEKMRSPMGTPTGSEIASPTLPSQVCSPTAAGLSMKRSIQRFLQKRKHRVQTTSPYNH*

**HbJAZ8.0a**

MNQRGRKEASDSAVPFGRQSSSFTFQPTSGRGQNPTQPTRQIEEEKETSDEQSPQHSQQLTIFYNGRVCVCDVTELQARAILLLASREMEENLRTPVGTPTGSEVASPSLPSPLCTPMAAGLSMKRSIQRFLQKRKHRVQAISPYNH*

**HbJAZ8.0b**

MRRNCNLELRLFPTSDQDHHRQTEASNEEQRQEQQITMFYNGSVCVADVTELQARAILMLASRTMEDNMRTNSSGSSSSSSSSGQLVSPTLASPLPVYSPNNNGLSMKMSLQSFLQKRNHRIQTTYPYNINRRPHACRVDH*

**HbJAZ8.0c**

MSRNCNLELSLFPTSDQYHRHQERRQQQQKLTIFYNGSVSVWDATEVQARAILMLAAQEMEDKMRITSTGPSSSSSSSLSSEQPVSPTLEPPPPPAYGPNNNGLSMKRSLQRFFQKRNHRIQATYPYNINRRPHACRVDHH*

**HbJAZ8.0d**

MSRNCNLELSLFPTSDQDHRHQERRQQQQKLTIFYNGSVSVWDATEVQARAILMLAAQEMEDKMRITSTGPSSSSSSSLSSEQPVSPTLEPPPPPAYGPNNNGLSMKRSLQRFFQKRNHRIQATYPYNINRRPHACRVDHH*

**HbJAZ9.0a**

MERDFMGLNSKESLPVVKEEVNSDGYKEIGFSKGSGIHWPFSNKVSALPHLNSFMVSQEDKTKRFLTDSSVSSGFLSISTADSFDPNQKQFMAEIQKSFNHDRQSGTHFTLTAYPVQHDVHSVHHPHDMKMFPVSNHASSISLSNPFFKNYYATSGQSTAGATAKPQLLGGIPVTTPQTILPTFGSVTGMMESCAKTSGSPAQLTIFYAGTVNVYNDISPEKAQAIMFLAGKDSSISSNMTQPNMTQPNNHIQEPSSKPIATDVSPVNHNVTTPPGSRLSSPLSVSSQTGSQSGSGSASTEEIMATKTAGVATTPVSKLDTPKLASAIGSVAATTLMPSAVPQARQASLARFLEKRKERVMSAAPYNLGKKSSESAIHNPME*

**HbJAZ9.0b**

MERDFMGLNSKEPLAVVKEEVNCDGYKEIGFSKSSGIHWPFSNKVSALPHLNSFKVSQEDKTKRLVSDSSLSPGFLSISTADAFDSNQKQFMAEIQKSFNHNRQSGTDFTLTAYPVQHDVHSVHHPRDMKMFPVSNHASSISLSNPFFKNYYAPSGQNVSGATVKPQLLGGIPVTTPQTILPTVGSVTGMMESCAIASGSPAQLTIFYGGTVNVYDDISPEKVQAIMFLAGQDSSISSNMALPKIQVHAPSSKPIATDVNPVNHNVTTPPCSRLSSPLSVSSQTGAQSGSGSTSTEEIMATKTTGVATTPVSKLDTPKLTSAMGSVAATTMMPSVPQARKASLARFLEKRKERVMSAAPYNMGKKSPESAMQNPIE*

**HbJAZ10.0a**

MSRATVELDFFGMEKQICANSHFPKFLNRQRSFRDIQSAISKINPELLKSVIASGSANQQSTPENGYHFDSKKSFSVPAAPKEQTPFPRLPVYYPLQRPALENPPQTAPLTIFYNGTVAVFDVPRDTAESILKLAENGFSKAVESTNQKQVLESLEGDLPIARRKSLQRFLEKRKERLTSLSPYACTPDCRS*

**HbJAZ10.0b**

MSRASVELDFFGMEKRISANSRFPKFLHRQRSFRDIQSAISKINPQLLKSVIASGSANQQTPENGHQLDSKKSFSVPSTPKEELNPFPPLPVYSPLQRPALENPPQTAPLTIFYNGTVAVFDVPRDKAETILKLAQNGISKFVESTNQKQLLGTLDGDLPIARRKSLQMFLEKRKERLTSVSPYACKSVCRL*

**HbJAZ11.0**

MEGESDSYREVKPKAGEDQLVPVNKSSLAATNGYDENLGSCKQGVLLQTNSSRNASMPTSGTNATIPTSDQLTIFYGGSVLVFDAIPAETAREIMLIAAAAAAAVKPADMKKAVSGSPAGGTPVLTRSPSLQSTTSALASPQTQLYSVHQGSSLCKLQAELPIARRHSLQRFFEKRRDRLCSKSPYPTPQAMKMAETTKPDFSAEVSPEAGCFGKTLAPEKEIQPKVTANLA*

**HbJAZ12.0**

MGSCKEGVLPWTNSSRPVSMATCGPNATVPTSDQLTIFYGGSILVFDTIPAEKVREIMLIAAAAAVAVKPADMKKAISGSPAGGTPVLTRSPSPQSSTSALPSPQAQVLPVHQGFSLRKLQAELPITRRHSLQRFFEKRRDRLCSKSPYPSPPAMKMAETIKPEFSAQVSPDAGCFGKPLAPEREIQPKVAANLA*

**HbJAZ13.0**

MSCRTPTRKEKELIGVEDDQNDNNPQVMASCLDKVAALKKKKHDQQRKMGFHHHQLTDDSRSQTLSMFRKYLCSKTQNSIVGKTIMEESEVIKKKHAATGRPLPPPKSGGHMPRQASLLEQKLLPGIRNEGMQSEYDGKSSAAQLTIFYAGAINVYDNVPADKAQAIMLLAGESSMSKPVAVELPRIETKKSPLHCSNLTSVCKLQPDLPIARKRSLQHFLEKRRRRIISKSPYSTPRAEHNKETKPRADDDGSNGDHSISLSPFPSRLGYFFPISANKGH*
