# Supplementary material for: Genome-Wide Identification and Characterization of the JAZ Gene Family in Rubber Tree (Hevea brasiliensis)
Source: Front Genet. 2019 May 1;10:372. doi: 10.3389/fgene.2019.00372 (PMC6504806; doi:10.3389/fgene.2019.00372)
Supplement: TABLE S3 — Evaluation of four rubber tree genome versions. [file Table_3.DOCX]

**Additional file 3**

**Table S3. Evaluation of four rubber tree genome versions.**

|  | Pootakham et al (2017) | Tang et al (2016) | Lau et al (2016) | Rahman et al (2013) |
| --- | --- | --- | --- | --- |
| Cultivar | BPM24 | Reyan7-33-97 | RRIM600 | RRIM 600 |
| Genome coverage | 68 x | 94 x | 155 x | 43 x |
| Number of scaffolds | 592,580 | 7,453 | 189,316 | 608,017 |
| N50 of scaffolds | 96.8 kb | 1.28 Mb | 67.24 kb | 2.97 kb |
