# Supplementary material for: Genome-Wide Identification and Characterization of the JAZ Gene Family in Rubber Tree (Hevea brasiliensis)
Source: Front Genet. 2019 May 1;10:372. doi: 10.3389/fgene.2019.00372 (PMC6504806; doi:10.3389/fgene.2019.00372)
Supplement: TABLE S4 — Gene accession numbers used in this paper. [file Table_4.DOCX]

**Additional file 4**

**Table S4. Gene accession numbers used in this paper.**

MdJAZ1: MDP0000187921; MdJAZ2: MDP0000301927; MdJAZ3: MDP0000193833; MdJAZ4: MDP0000135375; MdJAZ5: MDP0000174042; MdJAZ6: MDP0000718271; MdJAZ7: MDP0000173534; MdJAZ8: MDP0000173535; MdJAZ9: MDP0000889413; MdJAZ10: MDP0000565690; MdJAZ11: MDP0000891920; MdJAZ12: MDP0000452772; MdJAZ13: MDP0000244580; MdJAZ14: MDP0000243322; MdJAZ15: MDP0000871409; MdJAZ16: MDP0000285658; MdJAZ17: MDP0000241358; MdJAZ18: MDP0000757701; VvJAZ1: XM_002284819; VvJAZ2: XM_002262714; VvJAZ3: XM_003634778; VvJAZ4: XM_002272327; VvJAZ5: XM_002277733; VvJAZ6: XM_002277769; VvJAZ7: XM_002277916; VvJAZ8: CBI30922; VvJAZ9: XM_002277121; VvJAZ10: XM_002263220; VvJAZ11: XM_002282652; OsJAZ1: Os04g55920; OsJAZ2: Os07g05830; OsJAZ3: Os08g33160; OsJAZ4: Os09g23660; OsJAZ5: Os04g32480; OsJAZ6: Os03g28940; OsJAZ7: Os07g42370; OsJAZ8: Os09g26780; OsJAZ9: Os03g08310; OsJAZ10: Os03g08330; OsJAZ11: Os03g08320; OsJAZ12: Os10g25290; OsJAZ13: Os10g25230; OsJAZ14: Os10g25250; OsJAZ15: Os03g27900; AtJAZ1: At1g19180; AtJAZ2: At1g74950; AtJAZ3: At3g17860; AtJAZ4: At1g48500; AtJAZ5: At1g17380; AtJAZ6: At1g72450; AtJAZ7: At2g34600; AtJAZ8: At1g30135; AtJAZ9: At1g70700; AtJAZ10g: At5g13220; AtJAZ11: At3g43440; AtJAZ12: At5g20900; HbJAZ1.0: XM_021788791.1; HbJAZ2.0: XM_021780844.1; HbJAZ3.0: M_021807489.1; HbJAZ4.0: XM_021796925.1; HbJAZ5.0: XM_021783192.1; HbJAZ6.0: XM_021824535.1; HbJAZ7.0: XM_021793636.1; HbJAZ8.0a: XM_021834813.1; HbJAZ8.0b: XM_021834813.1; HbJAZ8.0c: XM_021793630.1; HbJAZ8.0d: XM_021793637.1; HbJAZ9.0a: XM_021832067.1; HbJAZ9.0b: XM_021831491.1; HbJAZ10.0a: XM_021790353.1; HbJAZ10.0b: XM_021817371.1; HbJAZ11.0: XM_021786280.1; HbJAZ12.0: XM_021835876.1; HbJAZ13.0: XM_021794816.1; SlJAZ2: Solyc12g009220; GhJAZ2: ES802380; SmJAZ3: KC864780; SmJAZ8: JQ936591; NtJAZ1: AB433896; NtJAZ2: AB433897; NtJAZ3: AB433898; AsJAZ1: DQ199647.1
